# Supplementary material for: MetaQTL: a package of new computational methods for the meta-analysis of QTL mapping experiments
Source: BMC Bioinformatics. 2007 Feb 8;8:49. doi: 10.1186/1471-2105-8-49 (PMC1808479; doi:10.1186/1471-2105-8-49)
Supplement: Additional File 7 — MetaQTL Package : jar file and tutorial. This Zip archive contains both the MetaQTL JAR file and the files of the tutorial. [file 1471-2105-8-49-S7.zip › org.inra.metaqtl/doc/org/thalia/bio/package-use.html]

Uses of Package org.thalia.bio


|  |  |  |
| --- | --- | --- |
| **Overview** \| **Package** \| Class \| **Use** \| **Tree** \| **Deprecated** \| **Index** \| **Help** | |  |
| PREV   NEXT | **FRAMES**    **NO FRAMES**     **All Classes** |  |


---


## **Uses of Package org.thalia.bio**

| Packages that use org.thalia.bio | |
| --- | --- |
| **org.metaqtl** |  |
| **org.metaqtl.adapter** |  |
| **org.metaqtl.algo** |  |
| **org.metaqtl.factory** |  |
| **org.metaqtl.main** |  |
| **org.metaqtl.util** |  |
| **org.thalia.bio** |  |
| **org.thalia.bio.entity** |  |
| **org.thalia.bio.entity.adapter** |  |
| **org.thalia.bio.entity.factory** |  |
| **org.thalia.bio.util** |  |

Classes in org.thalia.bio used by org.metaqtl

****IBioCross****
  
          Class Description Here

****IBioGenome****

****IBioLGroup****

****IBioLocus****

****IBioOntology****
  
          This interface defines some rules to handle a ontology.

Classes in org.thalia.bio used by org.metaqtl.adapter

****IBioCross****
  
          Class Description Here

****IBioGenome****

****IBioLGroup****

****IBioLocus****

Classes in org.thalia.bio used by org.metaqtl.algo

****IBioGenome****

****IBioLocus****

****IBioOntology****
  
          This interface defines some rules to handle a ontology.

Classes in org.thalia.bio used by org.metaqtl.factory

****IBioGenome****

****IBioLocus****

Classes in org.thalia.bio used by org.metaqtl.main

****IBioGenome****

****IBioLocus****

****IBioOntology****
  
          This interface defines some rules to handle a ontology.

Classes in org.thalia.bio used by org.metaqtl.util

****IBioGenome****

****IBioLocus****

Classes in org.thalia.bio used by org.thalia.bio

****IBioAdaptable****

****IBioAdapter****

****IBioAllele****
  
          Class Description Here

****IBioEntity****
  
          Class Description Here

****IBioGenome****

****IBioIndividual****
  
          Class Description Here

****IBioLGroup****

****IBioLocus****

****IBioOntologyTerm****
  
          This class defines a term of an ontology.

****IBioPopulation****
  
          Class Description Here

Classes in org.thalia.bio used by org.thalia.bio.entity

****IBioAdaptable****

****IBioAdapter****

****IBioAllele****
  
          Class Description Here

****IBioCross****
  
          Class Description Here

****IBioEntity****
  
          Class Description Here

****IBioGenome****

****IBioIndividual****
  
          Class Description Here

****IBioIndividualTree****
  
          Class Description Here

****IBioLGroup****

****IBioLocus****

****IBioOntology****
  
          This interface defines some rules to handle a ontology.

****IBioOntologyTerm****
  
          This class defines a term of an ontology.

****IBioPopulation****
  
          Class Description Here

Classes in org.thalia.bio used by org.thalia.bio.entity.adapter

****IBioAdapter****

****IBioEntity****
  
          Class Description Here

Classes in org.thalia.bio used by org.thalia.bio.entity.factory

****IBioEntity****
  
          Class Description Here

Classes in org.thalia.bio used by org.thalia.bio.util

****IBioGenome****

****IBioLocus****

---


|  |  |  |
| --- | --- | --- |
| **Overview** \| **Package** \| Class \| **Use** \| **Tree** \| **Deprecated** \| **Index** \| **Help** | |  |
| PREV   NEXT | **FRAMES**    **NO FRAMES**     **All Classes** |  |


---
